# A Mixed Reality Cognitive Orthosis to Support Older Adults in Achieving Their Daily Living Activities: Focus Group Study With Clinical Experts

**DOI:** 10.2196/34983

**Published:** 2022-07-20

**Authors:** Amel Yaddaden, Guillaume Spalla, Charles Gouin-Vallerand, Patricia Briskie-Semeniuk, Nathalie Bier

**Affiliations:** 1 École de réadaptation Université de Montréal Montreal, QC Canada; 2 Centre de recherche de l'Institut universitaire de gériatrie de Montréal Montreal, QC Canada; 3 Laboratoire Domus Université de Sherbrooke Sherbrooke, QC Canada; 4 Centre de recherche interdisciplinaire en réadaptation du Montréal métropolitain Centre intégré universitaire de santé et de services sociaux du Centre-Sud-de-l'Île-de-Montréal Montreal, QC Canada

**Keywords:** cognitive orthosis, mixed reality, older adults, daily activities, qualitative study, focus group, mobile phone, smartphone

## Abstract

**Background:**

Mixed reality is an emerging technology that allows us to *blend* virtual objects into the actual user’s environment. This can be realized using head-mounted displays. Many recent studies have suggested the possibility of using this technology to support cognition in people with neurodegenerative disorders (NDs). However, most studies have explored improvements in cognition rather than in independence and safety during the accomplishment of daily living activities. Therefore, it is crucial to document the possibility of using mixed reality to support the independence of older adults in their daily lives.

**Objective:**

This study is part of a larger user-centered study of a cognitive orthosis using pure mixed reality to support the independence of people living with NDs. This study aimed to explore (the difficulties encountered by older adults with NDs in their daily life to ensure that pure mixed reality meets their needs, (the most effective interventions with this population to determine what types of assistance should be provided by pure mixed reality technology, how the pure mixed reality technology should provide assistance to promote aging in place, and the main facilitators of and barriers to the use of this technology.

**Methods:**

We conducted a descriptive, qualitative study. A total of 5 focus groups were completed with occupational therapists who had expertise in the disease and its functional impacts (N=29) to gather information. Each focus group met once for a 1-hour period. All sessions were held over a 3-month period. A semistructured interview guide was used. All group interviews were audiotaped with the consent of each participant to facilitate the data analysis. We conducted inductive qualitative analysis in four stages using a thematic analysis approach: full transcription of the audio recordings, first-order coding of the transcribed data, second-order coding from the first-order code list, and data reduction and matrix development.

**Results:**

The results suggested that the main difficulties encountered by this population were in remembering to complete tasks, initiating the tasks, and planning the tasks. Several interventions are used to improve the independence of this population, such as prevention, simplification or facilitation, adaptation, and compensation. The use of pure mixed reality in older adults with NDs to promote independence and safety at home is promising and may respond to several clinical functions identified by the participants. Finally, pure mixed reality has good potential for use in this population and involves certain facilitators and obstacles, such as resources, technical aspects, and social considerations.

**Conclusions:**

The cognitive orthosis that will be developed in light of this study will act as a proof of concept for the possibility of supporting people with NDs using pure mixed reality.

## Introduction

According to the World Health Organization [1], in 2050, approximately 24% of the world population will comprise older adults compared with 14% in 2015. The aging of the population has led to an increase in the presence of neurodegenerative disorders (NDs), such as Alzheimer disease (AD) or mild cognitive impairments (MCIs), which has resulted in several challenges for this population and for society in general. Indeed, NDs have an important impact on the health care system, as it has been documented that 11% of people aged ≥65 years are living with an ND in Canada [2]. NDs rank fourth in the burden of disease, which is constantly increasing among caregivers and in the health care systems [3].

A way of reducing the burden on caregivers and on the health care system is to support aging in place by promoting home care through assistive technologies (ATs), comprising assistive, adaptive, and rehabilitative devices used to improve functioning and quality of life for people with disabilities or the population of older adults [4-7]. This would also support older adults’ desire to remain in their homes and thus live independently in the community for as long as possible [8]. To live independently in the community, a person must be able to perform basic activities of daily living (BADL), such as washing oneself and eating, as well as instrumental activities of daily living (IADL), such as managing finances, preparing meals, and taking medication. These activities are crucial for aging in place and maintaining the ability to live independently at home [9-11].

To assist in performing IADL, the AT should be able to spontaneously provide help to the person, including warning them of dangerous situations [12]. Such AT is called *intelligent* AT, which encompasses technologies that are able to capture and interpret the context in which the person is situated when performing an activity so that it requires the least amount of interaction and input from them [4,13,14]. To guide the development of such ATs and maximize their adoption by users, the *zero-effort technology* (ZET) principles have been proposed [12]. These principles involve designing technologies that (1) fit into the person’s environment and real-world setting, (2) compensate for the difficulties experienced by the person and match their residual capacities, (3) use intuitive interfaces, (4) reduce the caregiver burden, (5) protect the person's privacy and allow a sense of control, and (6) allow for adaptation and customization according to the person's preferences. Technologies that adhere to these principles involve minimal interaction with the user, allowing the user to focus on completing the task instead of on how to use the technology. In addition, it has been suggested that such technology presents better acceptability for people with moderate to severe cognitive impairments, such as older adults with NDs [15].

**Figure 1:**
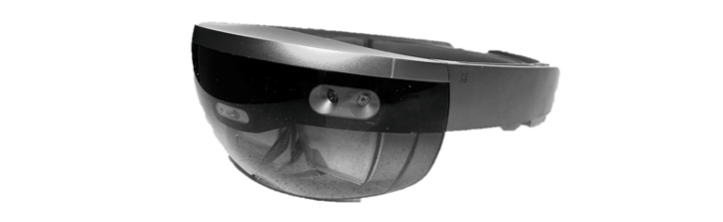
The head-mounted display.

Pure mixed reality realized with a head-mounted display (HMD; Figure 1) can, from a theoretical point of view, meet the ZET principles and has the potential to support older adults with cognitive deficits during their BADL and IADL. Pure mixed reality encompasses technologies that allow us to *blend* virtual objects in the actual user’s environment [16]. This is different from augmented reality (AR), in which holograms are overlayed onto the user’s environment [16]. To do so, the device uses various embedded sensors and computational capabilities to interpret the environment and understand the user’s context. This will allow the technology to intervene at any time and add virtual assistance to the environment without having to physically modify it. For example, the HMD can be used to scan the surroundings to detect where the user is in the home and what the user is doing. The HMD can also scan the surroundings to detect and recognize objects and provide assistance.

Only a few devices can currently realize pure mixed reality, the most advanced of which is the Microsoft HoloLens 2 [17,18]. One of the reasons for selecting an HMD rather than a mobile device such as a smartphone is that an HMD is, by definition, worn. It can continuously capture the environment, in contrast to a smartphone, which is carried. HMD will also always display the virtual information in front of the user’s eyes, in contrast to a smartphone, which needs to be pointed in the right direction. For these reasons, the use of an HMD can realize pure mixed reality efficiently and from a theoretical point of view, meeting the ZET principles.

To the best of our knowledge, the study of this technology to support older adults with NDs in achieving their daily activities is just beginning [13,14]. However, some researchers such as Blattgerste et al [13] are suggesting that their use in technologies to support older adults will increase because of the advantages it offers, such as providing audio and/or visual assistance and various possibilities for interactions (audio, gestures, and gaze). It is also expected that this technology will become more available in the years to come when prices will drop [13], and new designs will make HMD more usable and more versatile. To date, there has been positive reception from participants in studies conducted with HMD (based on AR and pure mixed reality) [13,19,20].

However, the types of difficulties for which older adults with NDs would require pure mixed reality assistance have not yet been documented in the literature. It is important to document these difficulties to be able to develop a prototype of an HMD based on the needs of the targeted population. Furthermore, it has not yet been specified in the literature how an HMD prototype can provide assistance to support the independence and safety of this population in BADL and IADL. Indeed, considering the limited literature on the subject, we need to understand what role the HMD could play in assisting this population and when to use such technology. It has been documented that the noncompatibility of technological advances with the needs of older adults is a major obstacle to their use and implementation; therefore, it is important to document their needs from the perspective of experts involved with this population, such as occupational therapists (OTs), to design a version that can later be tested with this population [21]. OTs are clinicians’ experts who have the knowledge and skills to assess older adults with NDs and provide appropriate ATs [22].

The objectives of this study were to document, from the experts’ perspective, (1) the main difficulties encountered by older adults with NDs in their daily life to ensure that the pure mixed reality meets their needs, (2) the most effective interventions for this population to determine what types of assistance should be given by the pure mixed reality technology, (3) how the mixed reality headset should provide assistance to respond to clinical purposes of promoting safety and independence at home, and (4) the main facilitators of and barriers to the use of this technology among this population to develop a version ready for laboratory testing.

## Methods

### The Research Design

This study is part of a larger user-centered design project on the design of a cognitive orthosis using pure mixed reality to support the independence of people living with NDs. Such an approach generally comprises four steps: exploration, ideation, generation, and evaluation [23,24]. This study reports the exploration phase, which aims to better understand users’ needs, motivations, and attitudes [22].

As various methods can be used to conduct the exploration phase [24], we selected a *descriptive inductive qualitative research design* to document the main difficulties of older adults with NDs and how assistance can be provided with pure mixed reality [25]. We collected the data through focus groups with OTs and experienced stakeholders to document their perspectives on an example of a mixed reality headset [20]. It has been documented that focus groups are relevant and appropriate for obtaining a detailed portrait of a phenomenon for which little literature exists [26]. The focus group method is particularly appropriate in this context to document the perspective of experts about the potential of using our first prototype to generate ideas because of group synergy [24].

### Participants and Recruitment Process

Invitations were sent to several OTs working in various clinical settings in specialized psychogeriatrics and experienced stakeholders of a local Alzheimer association. OTs are health professionals entitled to assess the needs of people living with cognitive impairments, determine the types of interventions that can ensure safety and increase their independence in IADL, and anticipate facilitators and obstacles to the use of new technologies, such as intelligent AT [27]. Experienced stakeholders, such as those involved in associations dedicated to older adults with NDs, are closely involved in the daily life and real environment of the person and are, therefore, able to specify the main difficulties encountered by this population, as well as predict effective interventions that work with older adults in their natural environment. The inclusion criterion was participants with at least 3 years of experience in involvement with older adults with NDs. There were no exclusion criteria for this study. Participants were divided into groups of 3 to 6 in accordance with the guidelines for this method [26].

### Ethics Approval

The Research Ethics Board of the Aging-Neuroimaging Research Ethics Committee of the *Centre* (*Intégré Universitaire de Santé et Services Sociaux*–*Centre-Sud-de- l’île-de-Montréal*) approved the project (CER VN 19-20-28*)*. The participants provided written and informed consent to participate in the study.

### Data Collection

Each focus group met once for 1 hour, and all sessions were held over a 3-month period. A semistructured interview guide was used (Textbox 1). Participants were asked to discuss three topics related to (1) the difficulties of older adults with NDs in everyday activities, (2) the effective interventions used to support the independence and safety of this population during meal preparation, and (3) their perspectives on the relevance of using the mixed reality headset with this population. A video describing the different features of the mixed reality headset was presented between topics 2 and 3. A member of the team (AY) acted as a facilitator and was responsible for asking questions and guiding discussions. Another member (GS) acted as an observer, took notes, and validated the discussion content with the group at the end of the discussion on each topic. All group interviews were audiotaped with the consent of each participant to facilitate the data analysis.

**Questions used to guide focus group discussions.**

- “In your experience, what are the main challenges faced by people in the early stages of neurodegenerative disorders in their daily activities at home?”
- “What are the main interventions you use with this clientele?”
- Presentation of a short video of the current version of our prototype and explanation of the parameters of use available to support the person during daily activities:
  - “How can our prototype be useful to support the daily living of this clientele?”
  - “Following the presentation of our prototype, do you think that such a tool can help elderly people with neurodegenerative disorders to improve their independence in daily living? What would you change to adapt this tool to the needs of your clients? Would you use such a tool with your clients, and if no, why?”

### Data Analysis

To ensure the validity of the data when using this type of method, words and facts were reported as accurately as possible following the focus group sessions, and we pursued data saturation. Data saturation is reached when there is sufficient information to replicate the study and when the ability to obtain additional new information has been attained so that further coding is no longer achievable. We attempted to remain close to our data, the words used, and the events described by recording the sessions and transcribing the entire verbatim [25]. We conducted inductive qualitative analysis in four stages using a thematic analysis approach [28]: (1) full transcription of the audio recordings, (2) first-order coding of transcribed data, (3) second-order coding from the first-order code list, and (4) data reduction and matrix development. To validate the data analysis, the lead author (AY) performed the coding. A list of codes was validated by an OT (PS) and a researcher from the team (NB) until a consensual integrated code list was obtained. The coding aimed to assign labels (codes) to relevant units of meaning, such as words, sentences, or paragraphs. After first-order coding, second-order codes were used to condense the data into different categories, which were then condensed into different major themes (third-order codes). Once the 2-step coding was completed, conceptual grouping matrices based on the major themes were developed to reduce the set of codes to a format that was more manageable and easier to conceptualize. All themes and matrices were validated by PS and NB.

## Results

### Overview

To obtain data saturation, a total of 24 OTs from different clinical settings, including a psychogeriatric-intensive functional rehabilitation unit, long-term care, day hospital, home support, and day center (n=10, 42% of OTs had >10 years of experience; n=14, 58% had 3-10 years of experience in geriatrics) were recruited, as well as 6 experienced stakeholders from a recognized Alzheimer association. They participated in a total of 5 focus groups. Table 1 presents the participant characteristics for each focus group session.

**Table 1:** Table of characteristics of participants.

Focus group and participant ID		Role	Gender	Clinical setting involvement
**Group 1**				
	P1	Occupational therapist	Female	Intensive functional rehabilitation unit
	P2	Occupational therapist	Female	Day hospital
	P3	Occupational therapist	Male	Intensive functional rehabilitation unit
	P4	Occupational therapist	Female	Day hospital Geriatric evaluation clinic
**Group 2**				
	P1	Occupational therapist	Female	Intensive functional rehabilitation unit Geriatric evaluation clinic
	P2	Occupational therapist	Male	Day center
	P3	Occupational therapist	Female	Intensive functional rehabilitation unit Home support
	P4	Occupational therapist	Female	Long-term care Day center
**Group 3**				
	P1	Occupational therapist	Female	Long-term care Day hospital
	P2	Occupational therapist	Female	Day hospital
	P3	Occupational therapist	Female	Intensive functional rehabilitation unit
	P4	Occupational therapist	Female	Home support Geriatric evaluation clinic
**Group 4**				
	P1	Experienced stakeholder	Female	Community-based association
	P2	Experienced stakeholder	Female	Community-based association
	P3	Experienced stakeholder	Female	Community-based association
**Group 5**				
	P1	Experienced stakeholder	Female	Community-based association
	P2	Experienced stakeholder	Female	Community-based association
	P3	Experienced stakeholder	Female	Community-based association

### Objective 1: Main Difficulties of Older Adults With NDs in Everyday Activities

Our first objective was to understand the main difficulties encountered by older adults with NDs in their daily lives to ensure that pure mixed reality meets their needs. The participants identified the main difficulties but also specified the factors influencing these difficulties (Table 2). Factors that influence the level of disability during activity performance were disease severity, social and professional support, and characteristics of the activity (newness, structure, and complexity).

**Table 2:** Participation of older adults with neurodegenerative disorders in everyday activities.

Disability types	Activities					
	Eating	Moving oneself	Washing oneself	Preparing meals	Managing medication	Managing finances
Difficulty in remembering to complete tasks	✓		✓			
Difficulty in initiating the tasks			✓	✓	✓	
Difficulty in remembering where they are in a task—what parts they already completed			✓	✓	✓	
Difficulty in planning a task		✓		✓	✓	✓

#### Main Type of Difficulties Encountered by Older Adults With NDs

According to the participants, the main difficulties encountered by this population were *difficulty in remembering to complete tasks*, difficulty in *initiating the tasks*, difficulty in *remembering* *where you are* in a task, and difficulty in *planning a task*. These difficulties could manifest in different activities, such as eating, getting around, bathing, preparing a meal, managing medication, and managing finances, as shown in Table 2.

For example, difficulty in *remembering to complete tasks* and *remembering where you are* in a task can occur during personal care, such as washing oneself or eating, as highlighted by 7% (2/29) of participants:

But they will start to have difficulty in washing, for example by forgetting and washing two or three times the same body part...hygiene, it is easy for them to wash a place, just a place and to forget or to think that they have already done everything.

P1, FG3, number 65

Well, I would tend to say that everything that happens at the level of nutrition in the kitchen, they are not able...as we were saying earlier, they are no longer able to get adequate nutrition, they think they have already eaten.

P2, FG4, number 34

According to the participants, difficulties in *initiating a task* comprise difficulty in getting into action, difficulty in thinking about doing a task, or difficulty in mobilizing without instructions or cues. Older adults with NDs can struggle to accomplish BADL, such as washing, getting dressed, or eating, as they have difficulties in thinking about what to do and putting these activities into action:

Well, lately, I’ve had a lot of very passive patients, because we weren’t even so much doing the task, but rather “initiating” it, e.g., thinking about washing, getting dressed, eating...just thinking of doing those was a challenge...

P1, FG1, number 67

I will also go uh it is very difficult for them at the level of putting into action to do things...without getting stimulated to do so.

P2, FG4, number 38

According to participants, difficulties in *remembering where you are* in a task appear mostly in the repetition of some steps of a task, as the person forgets the steps already taken and gets lost in the one that they perform:

Sometimes there's repetition of tasks too. I once had a lady who was doing her hygiene at the sink, she put on the deodorant, she washed again, she put the deodorant back on and then she asked me did I put it on or not? So, it's repetition, and she forgets the steps she did previously.

P2, FG2, number 29

And then in terms of repetition, especially, earlier I had a lady who was counting her money, an amount of money that I asked her to count for me.

P1, FG2, number 37

In contrast, difficulties in *planning the task* comprise difficulty in preparing all the elements necessary for the proper completion of the task without assistance. For example, a participant said the following:

A lot of difficulty in terms of preparation. Sometimes you really have to prepare the material for them, otherwise, nothing gets done.

P4, FG2, number 25

#### Factors Influencing Level of Difficulties

According to the participants, several factors influence the participation of older adults living with NDs in everyday activities, including *disease severity, social and professional support,* and *activity characteristics*. The *disease severity* influences the level of symptoms and disabilities, which has an impact on the accomplishment of activities, especially complex activities such as medication management, as presented in the following extract:

It also depends on the disease severity [...] But there could be an impact on all activities of daily living, but especially activities that are complex.

P2, FG3, number 34

*Social and professional support* are other factors that influence the level of disability in older adults living with NDs. Indeed, having the support of the family and adequate professional follow-up allows the person to have better needs management and a better quality of life. For example, having a proper medical follow-up with physicians and nurses influences the management of certain conditions that have a direct impact on the person’s capacities. According to a participant, an infection can have negative consequences on a person's ability to participate in everyday activities, as shown in the following extract:

I think that medication, nutrition, hydration are really important points, but I would also add that medical follow-up is very, very important to me because sometimes we know that a urinary tract infection can lead to delirium, can increase the symptoms, hallucinations, which means that for the person, it's a hell of a thing, she loses all her bearings. Uh, that can really have a major impact on the participation in daily activities. [...]

P1, FG4, number 40

Finally, *activity characteristics* such as newness, structure, and complexity directly influence the participation of older adults living with NDs. Indeed, new, unstructured, and complex activities such as meal preparation, financial management, appointment management, and medication management are more difficult to accomplish than routine, structured, and well-known activities. Newness is a factor that influences the level of difficulty in this population in many ways. It can involve difficulty in functioning in a completely new environment, difficulty interacting with new staff, difficulty in managing a new situation or task, and difficulty in having adequate judgment to react to an unexpected situation. In addition, this population has difficulty with activities without a specific structure, as explained in the following quote:

But they will start to have difficulty in washing without forgetting or going back to the same body part. Because the task has no structure imposed [...] e.g., hygiene, it's easy to make a place, just a place and forget or think that you have done everything. So, we'll see, so for the activities that are less structured, the difficulties will appear before.

P2, FG2, number 27

### Objective 2: Effective Interventions Used to Improve the Independence of Older Adults With NDs in Daily Activities

According to the participants, several interventions have been used to improve the independence of older adults with NDs. These interventions targeted the person’s abilities and task requirements or modified the environment to optimize the person’s safety, independence, and quality of life. According to the participants, these interventions had different goals, such as *preventing, facilitating, adapting,* and *compensating*, as shown in Table 3.

**Table 3:** Interventions used to improve the independence of older adults with neurodegenerative disorders in everyday activities.

Objective and target	Prevention or orientation	Facilitation or simplification	Compensation	Adaptation
Environment	Visiting the new environment before moving in Personalizing the new environment before moving in Involving the family	Providing physical assistance to initiate the activity (employee) Having a model that acts as an example Eliminating distractors	Caregiver doing the activity for them Using technologies to ensure safety during the task Using a pillbox or a dispill for medication management	Providing familiar elements Highlighting essential information
Person	Providing guidance	Providing feedback to continue the task Providing recall Providing verbal aid when achieving the task Graduating assistance when achieving the task	Stimulating the person to start the activity	Using a checklist Verbalizing the steps of the task when achieving the task
Activity	Providing directions and reference points	Reducing trip distances Preferring preauthorized payments and consolidating bank accounts and automated payments Using fewer ingredients and steps in recipes	Using preprepared meals	Using a timer to achieve meal preparation Establishing a routine

According to the study participants, *prevention interventions* mainly comprise upstream interventions to prevent undesirable situations. For example, in the case of institutionalization or delocalization, visiting the new environment and personalizing it before moving in is a relevant intervention for preventing disorientation, as well as involving the family and providing guidance, directions, and reference points. Regarding this, a participant pointed out the following:

Someone who doesn’t have any family, really, you hope that the residence in which she goes has an approach adapted to the elderly people and that...because if not, really, if she doesn't have any family to...even if it's only to personalize her room, to have her bedspread, a picture of her cat or anything. I think to guide her on a daily basis, to reassure her, to guide her...is really important.

P3, FG1, number 108

*Facilitation and simplification interventions* decrease the burden of actions by reducing the global complexity of activities. This can be accomplished by targeting the person, task, or environment. The goal of these interventions is to make the activity as simple as possible to make task accomplishment easier. For example, providing physical assistance to initiate the task or providing a model that acts as an example allowing for imitation are interventions that simplify the activity as they allow the person to skip the initiation and/or planification step of the task. For example, a participant said the following:

[...] when [they see] the attendants going to help the other clients, it is an example that is given, it saves them from doing the whole thread of planning the steps of the task and thinking about it.

P2, FG1, number 46

Providing feedback, verbal assistance, and visual cues are also *facilitation and simplification interventions* that facilitate task accomplishment as they allow the person to reduce their cognitive burden. According to the participants, using images instead of long sentences or having a list of preprogrammed steps are effective ways of providing facilitating assistance. Regarding this, 7% (2/29) of participants highlighted the importance of such interventions:

We’re going to try to simplify the task anyway.

P3, FG3, number 67

And we're always in the spirit of making it as simple as possible. One image, let's say, no more and not too many steps. Because we know that it won't be respected if there are too many.

P1, FG3, number 68

*Adaptation* interventions comprise modifying an element of the activity or the environment to allow the accomplishment of the task. According to the participants, this type of intervention differs from task simplification in that it involves adding elements and steps to the task instead of reducing it. For example, adding familiar elements or highlighting essential information in the environment, using a checklist to help planification, verbalizing the steps of the task when achieving it, using a timer to achieve some activities, or establishing a routine are effective interventions to support independence and safety according to participants. For example, a participant said the following:

I think that establishing a routine, organizing them in time and space, routine is a priority, uh, keeping them in their environment, huh, it’s really reassuring, secure for them, I could see that.

P4, FG4, number 165

Finally, according to the participants, *compensation* interventions comprise subtracting the steps of a task when elements are already performed by external help. For example, when the caregiver performs a task for them or when they stimulate the person to start the activity, they provide assistance that subtracts the initiation step of the task. Using technologies to ensure safety or using preprepared meals are also compensation interventions as they allow the person to skip steps within the tasks. For example, using a dispill and automatic recall allows a person to skip a few steps of the medication management activity, such as planning and organizing what pills to take. The participants said the following about the technologies and safety:

I think that when it comes to cognitive impairment, there is little potential for rehabilitation as such. I think that we go more into compensatory means. Really the services, for example the lifeline, the Safecook...

P2, FG2, number 143

The Safecook is like a box, a timer, you connect the stove to it, and you have to start the Safecook first before starting the stove. So, when you start the timer, it’s sure that after half an hour, it will be turned off even if the person hasn't closed it.

P1, FG2, number 15

### Objective 3: Opportunities for Use of a Mixed Reality Cognitive Orthosis

#### Overview

According to the participants, the use of pure mixed reality with older adults living with NDs to promote independence and safety at home was promising. Indeed, the participants identified three main clinical functions to which the mixed reality headset could respond: *assessment, assistance*, and *training*. *Assessment* comprises collecting information to evaluate a person. *Assistance* comprises providing explicit or implicit guidance to support task accomplishments. *Training* comprises providing information for developing new skills and abilities. These 3 functions could be responded to via 3 principal features of the mixed reality headset, including *detection, information storage and provision*, and *interactive* features (Table 4).

**Table 4:** Opportunities for use of a mixed reality cognitive orthosis to support older adults with neurodegenerative disorders to improve their independence and safety at home.

Microsoft HoloLens functionalities	Clinical functionalities		
	Provide assessment	Provide assistance	Provide training
Detection	Location and activity detection or recognition: Microsoft HoloLens can scan surroundings to detect where the user is in the home, what the user is doing, and whether the user needs emergency help	Object detection or recognition: Microsoft HoloLens can scan surroundings to detect objects and provide feedback through audio and video assistance Audio assistance involves assistance in finding objects (eg, remote control) and providing information about object functions Visual assistance involves assistance in providing the name of the object to the user, the name of the person through facial recognition to help social interactions, and warning symbols when the user is near danger (eg, stairs and stove)	N/A^a^
Information storage and provision	Task monitoring: Microsoft HoloLens can collect and store information on the user’s daily movements and activities to be collected by care providers: information on risky behaviors, information on routine, and number of omissions and errors during daily activities	Task support: Microsoft HoloLens can store information involved in specific daily routines and tasks to help the user perform them by providing information such as audio and video assistance Audio assistance involves assistance in providing daily reminders about upcoming appointment times and dates and in providing options (eg, dinner menu) Visual assistance involves assistance in providing pictograms of the steps of the task and a list of steps or choices	N/A
Interactive functions	N/A	Task support: Microsoft HoloLens can interact with the user using visual and auditory communication to help while the user is performing the task through audio and video assistance Audio assistance involves assistance in providing verbal feedback to the person and warning them in case of error and in mentioning the steps left to achieve the task Visual assistance involves assistance in providing symbols (eg, arrows, target, timer, and yes/no) to guide the person through medication, meal preparation, and leisure activities	Therapeutic guidance: Microsoft HoloLens can interact with the user to practice skills through guidance while the user is practicing the task, visual guidance and stimulation (eg, hemineglect), and guidance when the user is learning to use an object

^a^N/A: not applicable.

#### Detection Features

*Detection features* comprise the action or process of identifying the presence of objects in the environment or the user’s position or location. This allows location and activity recognition. Related to this feature, participants identified that the mixed reality headset may assist people in finding and correctly using objects by providing audio feedback to guide them through the environment or by providing information about object functions:

let me give you a basic example...let’s say someone who cleans his house and doesn’t remember what the products are for or confuse them...someone who sometimes takes detergent to wash the floor...The Mixed Reality Headset can help him to avoid that.

P4, FG4, number 345

Microsoft HoloLens detection features may also assist the person in finding and correctly using the objects by providing visual feedback such as an etiquette of the name of the object that appears in the virtual environment of the users. According to the participants, the mixed reality headset may also support social interaction through facial recognition by displaying the name of the people in front of the person. It can also ensure safety by providing visual assistance such as warning symbols when the user is near danger (stairs or stove). Related to these features, participants mentioned that using a stop sign symbol would send a clear signal to the user to avoid approaching a risky element:

I would see it more naturally with the concept of “forbidden,” for example: “You don’t do that”; “You don’t use the stove”; “You don’t go to the basement”; “You don’t go outside”; “You don’t use the stove”; “You don’t go to the basement”; “You don’t go outside”...You know, the things that are harder to compensate for in everyday life and that there isn’t someone there 24 hours a day to ensure safety.

P2, FG3, number 324

#### Information Storage and Provision Features

*Information storage and provision features* comprise accumulating information to anticipate the actions to take. For example, according to participants, the mixed reality headset may be useful for performing task monitoring to collect and store information on the user’s daily movements and activities and provide information on risky behaviors, routines, and the number of omissions and errors during daily activities. This can help in anticipating task support. Indeed, according to the participants, the mixed reality headset may be useful for storing information involved in specific daily routines and tasks to help the user perform them by providing information through audio and visual assistance. Audio assistance can be provided by providing daily reminders about upcoming appointments or by providing options and alternatives during activities (eg, dinner menu). Visual assistance can be provided through pictograms of the steps of a task or a list of steps or choices. Regarding this, a participant said the following:

Well, earlier when we were talking about the sequence, The Mixed Reality Headset can provide a visual pictogram...for example the person doesn’t know what step she is in the task...so what’s the next one? The visual aid could follow the steps and display a pictogram of the next one.

P1, FG3, number 378

#### Interactive Features

*Interactive features* comprise *communicating in real time with the user to respond to the three clinical functions*: assessment, assistance, or training. For example, according to the participants, the mixed reality headset can support task accomplishment by interacting with the user in real time to help through audio and visual assistance while the user is performing the task. Related to this functionality, participants mentioned that providing verbal feedback to the user and warning them in case of an error or mentioning the steps left to achieve the task are interactive functions that may be provided by the mixed reality headset. For example, a participant said the following:

When the person uses the dispill, the device may send a vocal message like “put the dispill back on the dining table”...it will help the person find it the next day.

P1, FG1, number 234

Providing symbols to guide the person (arrows, target, timer, and yes or no) through medication management, meal preparation, and leisure activities is also an interactive feature of the mixed reality headset, which may be useful, according to participants. For example, participants said the following:

If they’ve already set the destination in advance and have a real-time GPS with arrows and things that appear and detect cars...things like that, that could be interesting.

P3, FG3, number 382

Or for people walking around their house. So, someone who has a regular route is fine. But if sometimes when a construction gets lost, it is to wear a GPS headset so that he doesn’t get lost to go back home. [...]

P2, FG3, number 382

Indeed, integrating the GPS functions in the Mixed Reality Headset would be great.

P1, FG3, number 382

Interactive features allow the training of a person through *therapeutic guidance* as the mixed reality headset can interact with the user to practice skills. For example, the mixed reality headset can provide guidance while the user is practicing a task or when the user is learning to use an object. According to the participants, the mixed reality headset can also provide therapeutic guidance and stimulation to train the user’s visual scanning ability (eg, patients with hemineglect). Regarding this, participants underlined the following:

In the case the person doesn’t think to turn his head, you can provide visual cues that stimulate...or if the person is just scanning to one side, having an arrow or a light signal may help...

P1, FG3, number 392

### Objective 4: Facilitators and Obstacles Influencing the Use of the Mixed Reality Cognitive Orthosis by Older Adults With NDs

Overall, participants perceived the mixed reality headset as a technological tool with great potential for older adults with NDs. They identified several facilitators and barriers related to (1) *resources and technical aspects*, (2) *risks and ethical and social considerations*, and (3) *individual characteristics* that may influence the use of cognitive orthosis for this population, as described in Table 5.

**Table 5:** Facilitators and obstacles influencing the use of the mixed reality cognitive orthosis by older adults with neurodegenerative disorders to improve their independence and safety at home.

Category	Facilitators	Barriers
Resources	Human and professional resources Family involvement	Financial resources (costs) Maintenance and professional resources
Technical aspects	Possibility of connecting the device to the telephone Simplicity of use	Continuous wear of AR^a^ glasses Storage in the same place Appearance of the device not as a conventional eyewear
Ethical and social considerations	N/A^b^	Respect of privacy Social stigma
Individual characteristics	Ability to use and understand the usefulness of the device Presence of a need to use it Familiarity with technologies Interest in use	Lack of openness to using the device Disease severity Presence of sensory deficits (auditory and visual) Difficulty in finding the device Difficulty in remembering to put on the device
Risks	Technical resources (technical assistance)	Loss of visual, perceptual, and cognitive contact with the real environment Risk of nausea

^a^AR: augmented reality.

^b^N/A: not available.

#### Resources and Technical Aspects

In terms of resources, the involvement of the family and trained health professionals is a facilitator, as it allows for oversight of the use of the mixed reality headset. For example, 7% (2/29) of participants said the following:

And you need someone behind and all that too to facilitate the use, because there it’s someone else who’s going to put the cues, it’s someone else who’s going to know what to program, what are the activities that the person wants to do. So, there’s that side too, the setting up of the necessary cues too.

P3, FG2, number 458

And with a supervised part, a part that we come and do spot checks.

P4, FG2, number 458

According to the participants, in terms of technical aspects, the possibility of connecting the device to the smartphone may be a facilitator as it allows more flexibility and options of use. In addition, the simplicity of use has been shown to be an important factor that may facilitate the use of the device within this population. Regarding this, a participant said the following:

No, even the use of those glasses there, it really has to be user-friendly, don’t have to wonder too much about how it works. It must be very, very simple like keywords...

P3, FG2, number 54

Nevertheless, according to the participants, the continuous wear of the AR glasses can be a burden on the person as it can add to the heaviness of daily life. Some participants proposed limiting the use of this device to a few specific IADL only; however, others suggested that in this case, the device should always be stored in the same place, as, with the omission and memory loss issues of this population, the person may forget to wear the headset or forget where it is when doing an activity where they may need it. Regarding this, a participant said the following:

But I’m not sure, me practical, I don’t know how much technology is going to evolve, but how much you can ask a person to wear this 24 hours a day for all activities [...] I think wearing it maybe for an hour, the time of the activity, but you can’t expect someone to wear this all day.

P4, FG1, number 383

#### Risks and Ethical and Social Considerations

The appearance of the device, which is not similar to conventional eyewear, may be an important obstacle to the use of the mixed reality headset by this population. Indeed, as social stigma is a barrier to the use of this tool, according to the participants, it would be difficult to encourage this population to wear it in its current version. In addition, participants noted ethical considerations related to the aspect of privacy as the data collected by the mixed reality headset concerns the daily life of the person being assisted, who may feel watched. Regarding this, a participant said the following:

Yeah, because there’s an ethical issue behind that. There’s a question of privacy, of dignity, of the person as well, so they would have to be in, able to accept that their caregiver sees them uh, you know, at all times in their daily life. With that, I think, it would be to study.

P1, FG4, number 344

Some risks that could potentially be barriers to the optimal use of the mixed reality headset were reported by participants, including the risks of nausea, confusion, and loss of contact with the person’s actual environment while wearing the mixed reality headset.

#### Individual Characteristics

In relation to the individual characteristics of the person, several factors that can facilitate the use of the mixed reality headset were reported by the participants, such as being able to learn how to use the device, understanding its usefulness, and presenting the need to use it. Other factors such as familiarity with the technologies and interest in using them were shown to be important in facilitating the use of the mixed reality headset. In this regard, one of the participants said the following:

I think in several years, because we’re so exposed to technology and that kind of gadgetry, that if you start now, after I don’t know 10-15 years, I don’t know what your hope for timeline is, but more and more in we're going to use it so it’s going to become mainstream for us.

P1, FG1, number 438

Participants identified several barriers related to the characteristics of the person, such as the severity of the disability, which can have a significant impact on the person’s ability to use the tool. In addition, according to the participants, this population would be more likely to have sensory deficits, such as difficulties with sight or hearing, which could make it difficult to assist with daily activities using the mixed reality headset. This would also be the case for cognitive deficits; for example, if the person has difficulty finding the device or remembering its location, this can make it more difficult to use the mixed reality headset optimally. Regarding this, a participant said the following:

Just don’t be like me and not able to find your glasses! [laughs]. Because it’s a bad start if you need your glasses to find your glasses.

P1, FG1, number 409

## Discussion

### Principal Findings

The purpose of this study was to describe clinicians’ and experts’ perspectives on the potential of pure mixed reality to support independence and ensure the safety of older adults with NDs in daily life. More specifically, we aimed to document (1) the main difficulties encountered by older adults with NDs in their daily life to ensure that the pure mixed reality meets their needs, (2) the most effective interventions with this population to determine the types of assistance that should be given by the pure mixed reality technology, (3) how the mixed reality headset should provide assistance to respond to clinical purposes of promoting safety and independence at home, and (4) the main facilitators of and barriers to the use of this technology among this population to develop a version ready for laboratory testing. The results suggested the following: (1) the main difficulties encountered by this population are in remembering to complete tasks, initiating the tasks, remembering where they are in a task, and in planning the task; (2) several interventions are used to improve the independence of this population, such as prevention, simplification or facilitation, adaptation, and compensation interventions; (3) the use of pure mixed reality with older adults with NDs to promote independence and safety at home is promising and may respond to 3 clinical functions identified by the participants, including assessment, assistance, and training; and (4) pure mixed reality has good potential for use with this population, with certain facilitators and obstacles, such as resources and technical aspects, risks, ethical and social considerations, and individual characteristics.

### Comparison With Prior Work

Regarding the type of difficulties encountered (objective 1), our study showed that this population faced many challenges in the accomplishment of BADL and IADL, such as *eating, moving oneself, washing oneself, preparing meals, managing medication,* and *managing finances*. The main difficulties that this population may face in these activities are difficulties in *remembering to complete tasks*, *initiating the tasks, remembering where they are in a task* (what parts are already completed), and *planning the task.* Difficulties in initiating and planning are part of executive functions, which have been documented as one of the main cognitive functions affecting the dementia continuum [29-31]. Impairment in executive functions puts the person at risk of errors when faced with unexpected situations and when making an appropriate decision, which potentially affects independence and safety. In general, difficulties in remembering are part of the memory components documented to be affected in this population [32]. Memory impairments can affect learning abilities and a person's daily life from simple tasks (bathing and/or moving oneself) to more complex tasks (managing finances and/or preparing meals) [22] Memory and executive function impairments in this population are highly documented in the literature [33-37], and our results confirm the evidence. According to the participants, these difficulties would require specific assistance from the pure mixed reality technology to optimize independence and safety at home. Moreover, according to our results, many factors may influence the severity of difficulties in conducting these activities, such as disease severity, presence of social and professional support, and activity characteristics. *Disease severity* and *activity complexity* were shown to be determinant factors in the severity of difficulties that affect independence in IADL and BADL. Indeed, in the earlier stages of the ND continuum, such as MCI or early stages of AD, the difficulties are more inconspicuous, appearing only at the level of very complex activities that require the coordination of several steps. Furthermore, along the continuum, the difficulties are more visible and present in basic activities [38]. In short, pure mixed reality could be useful in the early stages of the disease to overcome memory- and executive function–associated difficulties encountered in the most complex activities of daily life, such as preparing meals and managing medication and finances.

The second objective of this study was to document the effective interventions that work with this population to determine the types of assistance that should be provided by pure mixed reality technology. Our study showed that according to the participants, clinical interventions can be effective when they target the person, activity, and/or environment, which is in accordance with models of occupational rehabilitation [39,40]. According to the participants, effective interventions aim to meet four main objectives: prevention, simplification or facilitation, adaptation, and compensation. Prevention interventions mainly comprise upstream interventions to specifically prevent undesirable situations, such as falling, getting lost, or causing a fire. In the literature, the prevention interventions described are generally aimed at delaying the onset of dementia and cognitive decline or reducing their incidences, such as promoting healthy lifestyle habits and providing education, intellectual stimulation, or early screening [41]. Nevertheless, our results focused on clinical practice in rehabilitation, as we mainly involved rehabilitation experts—OTs (24/29, 83%) and experienced stakeholders (6/29, 21%); thus, in our study, prevention interventions aimed to improve the person’s independence and safety and maintain their residual capacities, such as providing guidance, directions, and reference points to help the person prevent undesirable situations such as getting lost in a new environment. Several studies have shown that the use of this type of intervention allows a person to be more independent in their environment. For example, Spector et al [42] used a reality orientation board that displayed both personal and orientation information to provide some form of continuity for older adults with AD, which has been shown to be effective in preventing the risk of getting lost in the environment and in time. Simplification or facilitation interventions decrease the burden of actions by reducing the global *complexity* of activities. For example, according to participants, providing a model that acts as an example is a *facilitation* intervention that can make it easier to accomplish some activities, especially complex activities such as meal preparation. Few interventions of this type have been documented in the literature to support independence in IADL. Rousseau and Métivier [43] proposed an intervention based on *imitation* for emotional management. Adaptation and compensation interventions, as described in our study, such as using a pillbox or a dispill for medication management, highlighting essential information, stimulating the person to start the activity, using a checklist, verbalizing the steps of the task when achieving the task, or establishing a routine, are interventions that are widely documented in the literature as effective in optimizing independence and safety of older adults with NDs in their BADL and IADL [44-46]. Finally, the interventions proposed by the participants allow a better understanding of what types of assistance would meet the needs identified for this population, which would help computer scientists to better design features of HMD intended to help older adults with NDs accomplish BADL and IADL.

Our third objective was to determine the functions of the mixed reality headset that would be useful in assisting with the difficulties experienced by this population. Our study suggests that according to the participants, the detection, information storage or provision, and interactive features of a mixed reality headset can serve three main clinical functions: assessment, assistance, and training. In *assessment*, the mixed reality headset can detect or recognize the location and activity of the user and monitor them to provide data to clinicians about performance in daily activities. The use of sensor technologies has been shown to be promising in documenting the daily lives of older adults with dementia [47,48]. It has even been suggested that these technologies could be used to screen for NDs such as MCI or early AD [47]. More specifically, according to the participants, the mixed reality headset would be useful to document information on risky behaviors, routines of the person, and the number of omissions and errors during daily activities without the need for increased user interaction, which is particularly relevant to ZET principles [4,12,14]. Assistance was the clinical function that participants identified as the most useful for the mixed reality headset. Indeed, the mixed reality headset can scan surroundings to detect objects and provide assistance through visual and audio feedback to help the person accomplish their daily tasks. The mixed reality headset can also store information involved in specific daily routines and tasks to help the user perform them by providing audio and visual information such as daily reminders, options, instructions, pictograms, and a list of steps before the task or by interacting with the user during the task. These types of assistance have previously been documented in several other Assistive Technology Center design studies [49,50]; however, their use with older adults with NDs has limitations related to the nature of the proposed technology, as it often does not respect the ZET principles [12].

Finally, our last objective was to document the facilitators of and barriers to the use of the mixed reality headset among older adults living with cognitive impairments. Our study suggests that the mixed reality headset has good potential, with certain facilitators and barriers. Financial costs and maintenance resources were identified as the main barriers to the use of pure mixed reality. The affordability of technology is often identified by clinicians when discussing the use of technology with this population [21,51]. However, the use and early introduction of technology could delay the institutionalization of older adults with NDs for up to 8 months, when the technology is efficient and adapted to the person’s needs [52,53]. Thus, from a long-term perspective, it can be argued that the use of pure mixed reality at a more mature stage could support functional independence and, therefore, aging in place, as well as optimizing health care costs [52,54].

In terms of individual characteristics, some barriers to the optimal use of the mixed reality headset were reported by participants, including the risks of nausea, confusion, and loss of contact with the person's actual environment while wearing the headset. However, it has been documented that there is little to no *virtual reality sickness* in mixed reality or AR as there is no loss of contact with the real world; thus, it would be important to better inform people about this type of technology for optimized future adoption [55]. Social stigma may also be a barrier to the use of the mixed reality headset according to the participants, as it could be difficult to encourage this population to wear it in its current version. Considering the increasing technological advances in the domain, it will be possible to consider more conventional eyeglasses in the future [56]. In contrast, in earlier studies, the simplicity of use has been suggested to be an important factor that may facilitate the use of the mixed reality headset within this population, which reinforces the need to conduct usability testing in the future to document how to adapt and make the use of the device simpler, as required by the ZET principles [4,12,14,57]. In addition, other factors documented in our study, such as familiarity with the technologies and interest in using them, are shown to be important in facilitating the use of the mixed reality headset [21]. These factors are indeed part of the Technology Acceptance Model, which is a key model for understanding predictors of human behavior toward potential acceptance or rejection of the technology [58]. Considering the growing increase in technology use by older adults [51,59,60], it is possible to predict that the mixed reality headset will be democratized and implemented as an AT for home support services in the future.

However, we acknowledge that the current development of mixed reality headsets has several limitations that could prevent the democratization of such technology to people with cognitive disorders and, more widely, to the general public for several years [13]. Despite positive preliminary studies, the high weight of this device might affect the user’s experience [13,61,62]. Moreover, immersion is not optimal because of the small field of view of the screens [13]. The high price of the device is also an obstacle to its mass adoption [13]. The design of 3D graphical user interfaces and interactions is also important and should be carefully considered [63]. The literature suggests paying attention to the specific needs of older people with cognitive disorders during the design process because of their cognitive, perceptual, or physical limitations [13,64-68], which is supported by the conclusions of this study. For example, it is suggested to limit cognitive overload by limiting possible options [66], and the size of icons should be large enough as small targets might be difficult to reach [68].

If these issues are addressed, mixed reality could have several benefits, as well as limitations, because of their nature compared with other AT. In contrast to smart environments, mixed reality headsets do not require any modifications to the user’s environment. In contrast, the user is required to wear the device to receive assistance. This might be inappropriate in certain situations, for instance, in the case of night wandering [69]. In contrast to smart environments, which are stationary, mixed reality headsets can deliver assistance at any time and place. However, they cannot act directly on the user’s environment, for example, to prevent the evolution of a dangerous situation. Embedded technologies, such as the Cognitive Orthosis for Cooking, can turn off power to the stove if unsafe use by a person with a cognitive disorder is detected [70,71]. The mixed reality headset will also not be able to monitor the user’s health, in contrast to body sensors or some smart environments [66].

Smartphones are popular devices that offer an alternative to delivering AR or mixed reality apart from headsets [13]. This technology is inexpensive and socially accepted [13]. However, smartphones offer less mobility than a headset, as the user needs to hold them with one hand and point the device in the direction where the assistance will be located in the space [72]. A mixed reality headset provides assistance in front of the user, allowing the user to keep their hands free [72]. Headsets also offer a more immersive experience [72]. Finally, projectors can be used to free the user from wearing a device and to use both hands [13]. However, in most cases, projectors are stationary [13] and, consequently, cannot offer assistance at any time or place.

### Future Directions

Several future research paths can be suggested to continue advancing knowledge about the potential of mixed reality with individuals with NDs. First, observational studies can be undertaken to refine the users’ requirements based on the directions proposed by health care professionals. Second, ZET mixed reality headset prototypes could be developed to be useful for the target population by following a user-centered design approach. In particular, optimal interactions and graphical user interfaces should be explored. Regular usability testing with users should also be undertaken to maximize the usefulness of the prototype. Third, specific mixed reality ZET principles could be developed for this population by completing the already existing guidelines [65]. Fourth, coupling mixed reality headsets with both sensors to monitor the user’s health and smart environments to manage critical situations could be explored. Finally, evaluating whether those results can be transposed to other populations, such as people with traumatic brain injuries or children with neurocognitive development disorders, could help in generalizing the applicability of mixed reality to other populations in need of ATs.

### Strengths and Limitations

This study had some limitations. First, >75% of our participants were female, which may represent a gender issue. However, this reflects a reality in health care settings where women represent most health care professionals to which our participants belonged. Second, participants were recruited from clinical settings within a single city, which may limit the generalizability of the results. However, our focus groups were homogenous [73], as recommended by qualitative research guides, as all participants were OTs or experienced stakeholders from diverse settings in psychogeriatrics. This diversity allowed for in-depth documentation of our assumptions regarding the functional profiles of older adults with NDs across the continuum of care. Third, data saturation was noted as early as in the fourth focus group, reinforcing the credibility of the results obtained from the analysis. Finally, this study does not directly document the end user perspective but instead involves them indirectly, which is a limitation to the applicability of the results currently. However, we decided to initiate the first step of the user-centered design cycle (exploration) by involving clinical experts as our rationale was that older adults living with NDs could have had difficulty answering our research questions because of a lack of abstraction abilities required to discuss an intangible topic [74,75]. It has been previously documented that the low maturity of technology is a barrier to the initial intention for use and may result in the rejection of the prototype in the future. Therefore, we took the possible obstacle into consideration when choosing our first design step [76]. Our intention was to document the needs of older adults with NDs from the perspective of experts to design a version that can later be tested by them. Our next step will be to design a prototype that meets the recommendations identified in this study (ideation and generation) and then test its usability with older adults living with NDs and their caregivers (evaluation).

### Conclusions

This study aimed to describe experts’ perspectives on the potential of pure mixed reality to support independence and ensure the safety of older adults living with NDs. The results suggest that a mixed reality cognitive orthosis may help older adults with NDs face difficulties in everyday activities, such as *remembering to complete tasks*, *initiating the tasks, remembering where they are in a task* (*what parts are already completed*), and *planning the task.* Thus, the use of mixed reality cognitive orthosis in older adults living with NDs to overcome these difficulties and promote independence and safety at home is promising and may respond to several clinical functions identified by the participants, including assessment, assistance, and training. Finally, the mixed reality headset has good potential for use with older adults with NDs, with certain facilitators and limits. Future studies should address usability testing in this population to develop a usable and implementable prototype to support aging in place.

## Abbreviations

AD: Alzheimer disease
AR: augmented reality
AT: assistive technology
BADL: basic activities of daily living
HMD: head-mounted display
IADL: instrumental activities of daily living
MCI: mild cognitive impairment
ND: neurodegenerative disorder
OT: occupational therapist
ZET: zero-effort technology
